# Ferrous versus Ferric Oral Iron Formulations for the Treatment of Iron Deficiency: A Clinical Overview

**DOI:** 10.1100/2012/846824

**Published:** 2012-05-02

**Authors:** Palacios Santiago

**Affiliations:** Palacios Institute of Woman's Health, Antonio Acuña 9, 28009 Madrid, Spain

## Abstract

Iron deficiency anaemia represents a major public health problem, particularly in infants, young children, pregnant women, and females with heavy menses. Oral iron supplementation is a cheap, safe, and effective means of increasing haemoglobin levels and restoring iron stores to prevent and correct iron deficiency. Many preparations are available, varying widely in dosage, formulation (quick or prolonged release), and chemical state (ferrous or ferric form). The debate over the advantages of ferrous versus ferric formulations is ongoing. In this literature review, the tolerability and efficacy of ferrous versus ferric iron formulations are evaluated. We focused on studies comparing ferrous sulphate preparations with ferric iron polymaltose complex preparations, the two predominant forms of iron used. Current data show that slow-release ferrous sulphate preparations remain the established and standard treatment of iron deficiency, irrespective of the indication, given their good bioavailability, efficacy, and acceptable tolerability demonstrated in several large clinical studies.

## 1. Introduction

Iron deficiency anaemia (IDA) is the condition in which there is anaemia due to a lack of iron. IDA develops when available iron is insufficient to support normal red cell production and is the most common type of anaemia [1].

According to the World Health Organisation (WHO) [2], iron deficiency is the most common form of malnutrition in the world, affecting around 2 billion people worldwide, which corresponds to 25% of the population globally. Iron deficiency is highly prevalent in developing countries where it represents a major public health problem, but it is also common in Western countries, particularly in populations such as infants, young children, women with heavy menses, and pregnant and puerperal women [3]. Women are at high risk of developing IDA during pregnancy due to increased iron requirements [4, 5]. Iron deficiency anaemia independently increases morbidity and mortality [6]. In France, a large epidemiological study (SUVIMAX trial) [7] has shown that approximately 93% of women have insufficient dietary iron intake and 23% of women of reproductive age are iron deficient, 4% of whom are anaemic.

Common causes of iron deficiency include inadequate intake of dietary iron, inadequate iron utilisation during chronic and inflammatory diseases, impaired iron absorption, or excess iron loss. In the vast majority of cases, the cause of iron deficiency anaemia results in an anaemia that is both avoidable and reversible by increasing iron supplementation or reducing iron loss.

Iron is essential for oxygen transport and cell growth and survival. The typical adult human body contains an average of 3.5 g of iron (approximately 4 g for males and 3 g for females). Most of the iron within the body is used in haemoglobin (2.1 g). A small amount is devoted to cellular protein synthesis (myoglobin, cytochromes) or circulates through plasma bound to transferrin [7]. Iron homeostasis is closely regulated via intestinal absorption and by recycling of iron already present in the body. This element has the particularity that once absorbed, there is no physiologic mechanism for its excretion from the body. Only 1 mg of iron is lost per day by males and 2 mg by menstruating females (through blood and mucosal epithelial cell loss).

To maintain adequate supplies of iron for heme synthesis, 20 mg of iron is recycled daily, going from senescent red cells that are removed from the circulation to new cells in the bone marrow [8]. Iron from older cells is loaded onto transferrin by macrophages for delivery to the bone marrow. The diet provides 10–20 mg per day of iron requirement, as heme (mainly in red meat) and nonheme (white meat, vegetables, and cereals). Healthy adults absorb approximately 10 to 15% of this iron in their diet, but absorption is influenced by the body's iron stores, the type of iron in the diet (heme and nonheme), and other dietary factors that may increase or reduce the absorption of iron. Heme iron is absorbed very efficiently by the body whereas only 1 to 7% of nonheme iron is absorbed [9]. Because nonheme iron is present mainly as ferric iron in food, it must be reduced to the ferrous and divalent form (Fe^2+^) prior to uptake by intestinal enterocytes [10]. Around 1-2 mg/day of additional dietary is needed to balance losses in the urine, sweat, and stools. The hormone hepcidin regulates iron homeostasis by controlling ferroportin-mediated release of iron from enterocytes and macrophages [11].

For the treatment of iron deficiency anaemia, current guidelines recommend the dose of 60 to 120 mg of elemental iron of ferrous sulphate per day for a minimum duration of 3 months in adolescents and adults, including pregnant women [12]. Given that it is difficult to satisfy the increased iron requirement during pregnancy by dietary means [13], most international health organisations [14] and national authorities recommend oral iron supplementation during pregnancy. The recommended dose for the prevention of iron deficiency anaemia during pregnancy is generally 60 mg of elemental iron per day to be taken during pregnancy and for the 6 months postpartum for pregnant women who did not begin iron supplementation in the second trimester of the pregnancy [15]. International organisations including WHO and UNICEF recommend oral iron supplementation for young children and adolescents in countries where the prevalence of anaemia in the population is over 40% [16].

In the case of iron deficiency anaemia, once the underlying cause has been identified and treated, iron replacement therapy is necessary to correct haemoglobin levels and replenish iron stores. From a practical point of view, the oral route is the first choice to replace iron stores as this allows the normal mechanism of absorption to be used and thus may prevent complications and the risk of iron overload, such as is reported with intravenous iron administration, in addition to being an inexpensive and effective treatment. Many oral iron preparations are available, the most frequently used being ferrous sulphate (FS) and ferric preparations with an iron polymaltose complex (IPC). Most of these preparations vary in their bioavailability, efficacy, side effects, and cost. Here we review the data available in the literature with regard to the efficacy and tolerability of ferric and ferrous preparations currently used in clinical practice, and especially sustained-release FS versus IPC which are among the most prescribed iron formulations in the world.

## 2. Bioavailability and Therapeutic Efficacy of Bivalent and Trivalent Iron Preparations

The iron-containing preparations available on the market vary widely in dosage, salt, and chemical state of iron (ferrous or ferric form) contained in the preparation, as well as the galenic form (quick and prolonged release). However, in clinical practice bivalent iron salts such as FS, ferrous gluconate, and ferrous fumarate are more widely used and are preferred over ferric iron preparations [17, 18], as recommended by the WHO [19]. FS preparations usually present good bioavailability (between 10 and 15%), while bioavailability of iron ferric preparations is 3 to 4 times less than that of conventional FS [20]. This is due to the extremely poor solubility of ferric iron in alkaline media and the fact that ferric iron needs to be transformed into ferrous iron before being absorbed (Table 1). Among ferrous preparations, FS remains the established and the standard treatment of iron deficiency given its acceptable tolerability, high effectiveness, and low cost. 

Advances in oral preparation have led to the development of prolonged-release preparations with new galenic formulations that may improve gastrointestinal tolerability and enhance the bioavailability. Among these compounds, the most studied and prescribed is Tardyferon^®^, a prolonged-release ferrous sulfate tablet containing 80 mg of elemental iron. In this product a polymeric complex surrounds Fe^2+^ ions forming a matrix that controls the availability of Fe^2+^ ions to the individual sections of the gastrointestinal tract in conformity with their absorptive capacity. After its absorption, iron levels in the blood reach a maximum after about 7 hours and remain elevated for 24 hours. In a study conducted by Kaltwasser et al. [21], the bioavailability of Tardyferon^®^ was compared to that of a quick-release ferrous ascorbate preparation in 18 healthy phlebotomized volunteers, using a stable ^54^Fe iron isotope. The study did not find any difference in iron intestinal absorption measured on day 21 between the two preparations. Moreover, after two months of treatment, haemoglobin levels increased to approximately baseline values in both treatment groups.

Maltofer^®^/Ferrum Hausmann^®^/Ferranina^®^ is a trivalent oral iron (100 mg of element iron) coupled with sugar complexes (IPC). This structure is believed to give the ferric iron compound a better stability and portability of ferric iron ions through the intestinal mucosa under physiological conditions, compared to conventional ferric compounds [22]. While some reports indicated that the availability of iron from IPC for haemoglobin synthesis is comparable to that of conventional ferrous salts such as FS [23–25], many studies have reported poor effectiveness of iron from ferric polymaltose complex [26–30]. Mehta was the first to publish individual clinical case reports of patients failing to respond to IPC [25, 26]. In 2003, Mehta [31] published a report of 27 patients with iron deficiency anaemia who failed to respond to IPC given for 4 to 52 weeks and showed that the same patients responded to the administration of ferrous fumarate for 4 to 13 weeks. Similar data was obtained by Ruiz-Argüelles et al. [30] who showed that among 240 patients diagnosed with iron deficiency anaemia in his institution and treated with oral IPC, 75 (31%) failed to respond. Median haemoglobin levels when the patients were referred for the study after being given oral IPC was 10.3 g/dL. After administration oral ferrous fumarate during periods ranging from 1 to 14 months, haemoglobin levels rose to a median of 12.5 g/dL (*P* < 0.01). 

Kaltwasser et al. [32] also compared trivalent versus bivalent preparations and showed a significant difference in the bioavailability of ^59^Fe III hydroxide-polymaltose compared to that of ^59^Fe labeled-bivalent iron preparations (ferrous ascorbate or a quick-release FS preparation). Intestinal iron absorption in the fasting state, as measured by ^59^Fe whole body retention and simultaneous estimation of plasma iron tolerance curves, was low for the Fe III complex (1.2 ± 0.1%) as compared to ferrous ascorbate (43.7 ± 7.1%). After a meal, the absorption of the divalent preparation was not affected, whereas that of the Fe III complex increased to 8.8 ± 4.7%. However the daily increase in haemoglobin concentrations after an equivalent therapeutic dose of 100 mg elemental iron during 28 days was greater for the divalent preparations compared to the Fe III hydroxide-polymaltose complex (1.1 ± 0.3 g/L versus 0.68 ± 0.2 g/L). Similar observations were reported by Malhotra et al. [33] and Heinrich et al. [34] on the poor bioavailability of the trivalent preparations. Nielsen et al. [35] found no haemoglobin increase in 9 patients receiving 100 to 300 mg of ferric polymaltose complex on an empty stomach during a 4-week treatment period. On the other hand, subsequent treatment with ferrous sulphate (100–200 mg Fe/day) resulted in a significant increase of haemoglobin (0.15–0.23 g/dL per day). In another study conducted by Nielsen et al. [36], 33 patients with chronic haemorrhagic iron deficiency anaemia (Hb <12 g/dL, serum ferritin <12 *μ*g/dL) received Tardyferon^®^ (1 tablet/day) over 6 to 10 weeks. Significant increases in haemoglobin and ferritin concentrations were observed within this period (mean Hb increased from 10.2 ± 1.6 to 12.5 ± 1.5 g/dL; ferritin, from  9 ± 11  to 31 ± 23 *μ*g/L), indicating that specific prolonged-release iron preparations may provide relatively high iron bioavailability and are effective in the treatment of iron deficiency anaemia, even in the case of chronic haemorrhage. Only one blinded, double-dummy randomised trial conducted by Langstaff et al. [37] compared the efficacy and tolerability of IPC preparations (Ferrum Hausmann^®^, 200 mg elemental iron/day) to standard FS preparations (180 mg elemental iron/day). Both were given to 126 adult patients during 9 weeks. FS resulted in a significantly higher increase in haemoglobin levels compared to Ferrum Hausmann^®^ at 3 and 6 weeks. At week 9 the difference between both groups was not statistically significant.

Other findings concerning the lack of efficacy of IPC versus FS were reported in studies on risk groups for anaemia as children, pregnant women, and the elderly. Two large randomised trials assessed the efficacy and tolerability of iron polymaltose complex versus FS, in the treatment of iron deficiency anaemia in children. The first study [38] was conducted in 118 children who were randomised to receive either oral IPC or oral FS at an equal dose of 6 mg/kg/day, on an empty stomach for one month. The increase of haemoglobin one month after the start of the therapy was significantly higher in the group of children having received FS (9.44 ± 0.67 g/dL) compared to the group of patients treated with IPC (8.67 ± 0.73 g/dL). In addition, approximately 21% of the children in the IPC group had decreased haemoglobin levels after treatment compared to baseline values. Lack of effectiveness of IPC in children was also reported by Haliotis and Papanastasiou in 100 anaemic children receiving 4 mg/kg/day of iron for a 2-month treatment period [39]. The efficacy of IPC in the treatment of iron deficiency anaemia during pregnancy has not been well established, and conflicting results have been reported [40–42]. On the other hand, a daily dose of 80 mg of elemental iron contained in one tablet of the preparation Tardyferon^®^ was shown to be sufficient for recovery of iron reserves within the puerperium period, as shown by Mára et al. [43]. In elderly patients with iron deficiency, similar findings of poor effectiveness of IPC were reported by Sanders [44].

## 3. Tolerability of Ferrous versus Ferric Iron Preparations

Side effects of oral iron therapy are a common problem in the treatment of patients with iron deficiency. Gastrointestinal disturbances such as nausea, heartburn, pain, constipation, and diarrhoea are the most commonly reported side effects, irrespective of the type of iron preparation. This occasional intolerance is usually viewed as a limiting factor for oral iron therapy, as it may impact patient compliance. The incidence of the gastrointestinal side effects seems to be generally associated with the use of unnecessary high doses of iron as reported by several authors [45, 46]. High iron doses may be necessary in the case of anaemia.

Incidence of gastrointestinal side effects has been shown to be lower with controlled-release iron formulations compared to conventional ferrous salt preparations in three large controlled randomised studies [47–49]. In such formulations, iron is released at a slower rate because of action of gastric acid on the matrix containing FS, thus reducing the bolus load of iron into the gastrointestinal tract, hence producing fewer side effects. In a systemic review of 106 studies published up to 2008, including data from 10,515 patients treated with various oral iron preparations, Manasanch et al. [50] found that sustained-release FS (Tardyferon^®^) had a statistically significant lower incidence of gastrointestinal events (3.7%) compared to other FS preparations (31.6%), ferrous fumarate (44.8%), and to preparations containing ferric iron such as iron protein succinylate (7.0%). The results of this study demonstrated clearly that sustained FS release preparations are better tolerated than other preparations including ferric iron preparations.

In the Langstaff et al. [37] study mentioned above (bioavailability/efficacy section) that compared IPC preparations and standard FS preparations given at equivalent therapeutic doses to 126 patients, adverse events were reported in 12 patients (22%) treated with Ferrum Hausmann^®^ and 14 (25%) patients in the standard FS group. The majority of events were gastrointestinal in nature: constipation was reported in 18% of patients in the Ferrum Hausmann^®^ group versus 11% in the standard FS group and abdominal pain in 10% of patients in the Ferrum Hausmann^®^ group versus 18% in the standard FS group.

## 4. Conclusion

Oral iron supplementation is the standard treatment for patients with iron deficiency. Ferrous salts and in particular prolonged release FS preparations are the treatment of choice given their high effectiveness, acceptable tolerability, and low cost. Preparations with iron III hydroxide polymaltose generally display poorer bioavailability and their clinical efficacy is yet to be established. The claimed superiority of ferric iron preparations over sustained-release ferrous sulphate preparations is also questionable. Only preparations for which efficacy and tolerability have been proven should be used in the treatment of iron deficiency.

## Figures and Tables

**Table 1:** Differences between bivalent and trivalent oral iron preparations.

Iron supplement	Comments
Bivalent	
Ferrous fumarate (Fe^2+^)	More adverse effects if not in a prolonged-release formulation
Ferrous gluconate (Fe^2+^)	
Ferrous sulphate (Fe^2+^)	
Ferrous glycine sulphate (Fe^2+^)	

Trivalent	Poorer absorption
Iron protein succinylate (Fe^3+^)	More expensive
Iron polymaltose complex (Fe^3+^)	A greater number of intakes
